# Resource Needs for Adolescent Friendly Health Services: Estimates for 74 Low- and Middle-Income Countries

**DOI:** 10.1371/journal.pone.0051420

**Published:** 2012-12-27

**Authors:** Charlotte Deogan, Jane Ferguson, Karin Stenberg

**Affiliations:** 1 Department of Public Health Sciences, Karolinska Institutet, Stockholm, Sweden; 2 Department of Maternal, Newborn, Child and Adolescent Health, World Health Organization, Geneva, Switzerland; 3 Department of Health System Financing, World Health Organization, Geneva, Switzerland; University of Cape Town, South Africa

## Abstract

**Background:**

In order to achieve Millennium Development Goals 4, 5 and 6, it is essential to address adolescents’ health.

**Objective:**

To estimate the additional resources required to scale up adolescent friendly health service interventions with the objective to reduce mortality and morbidity among individuals aged 10 to 19 years in 74 low- and middle- income countries.

**Methods:**

A costing model was developed to estimate the financial resources needed to scale-up delivery of a set of interventions including contraception, maternity care, management of sexually transmitted infections, HIV testing and counseling, safe abortion services, HIV harm reduction, HIV care and treatment and care of injuries due to intimate partner physical and sexual violence. Financial costs were estimated for each intervention, country and year using a bottom-up ingredients approach, defining costs at different levels of delivery (i.e., community, health centre, and hospital level). Programme activity costs to improve quality of care were also estimated, including activities undertaken at national-, district- and facility level in order to improve adolescents’ use of health services (i.e., to render health services adolescent friendly).

**Results:**

Costs of achieving universal coverage are estimated at an additional US$ 15.41 billion for the period 2011–2015, increasing from US$ 1.86 billion in 2011 to US$ 4,31 billion in 2015. This corresponds to approximately US$ 1.02 per adolescent in 2011, increasing to 4.70 in 2015. On average, for all 74 countries, an annual additional expenditure per capita ranging from of US$ 0.38 in 2011 to US$ 0.82 in 2015, would be required to support the scale-up of key adolescent friendly health services.

**Conclusion:**

The estimated costs show a substantial investment gap and are indicative of the additional investments required to scale up health service delivery to adolescents towards universal coverage by 2015.

## Introduction

In a growing number of countries, a demographic transition is occurring. As children survive the dangers of childhood illnesses and move into the second decade of their lives, there is a bulge in the adolescent band of the population pyramid. [1] The current cohort of young people worldwide is the largest it has ever been. In 2010, the International Year of Youth, there were 1822 million young people 10–24 years of age – representing one quarter of the world’s population. [2] Four out of five young people live in less developed countries, and represent up to one third of those countries’ populations. The health of young people has been largely neglected in global public health because this age group is perceived as healthy. [3] However, every year, 2.6 million young people die. Most of these deaths are preventable. Some 97% of these deaths occur in low- and middle-income countries. [4] Over the past 50 years, mortality rates in all age groups have declined. However, mortality among young people has decreased less than in other age groups, overtaking childhood mortality in some high-income countries. Moreover, morbidity accounts for a greater proportion of burden of disease in this age group than mortality.[4]–[5].

In order to achieve Millennium Development Goals (MDGs) 4 (reduce child mortality), 5 (improve maternal health) and 6 (combat HIV/AIDS, malaria and other diseases), it is essential to address adolescents’ health. While different sectors need to work together, one of the roles of the health sector is to expand the coverage and improve the quality of health services for adolescents (especially those who are more likely to face health and social problems) in order to achieve clearly defined health outcomes.[6]–[7] The scaling-up of services requires adequate and reliable funding and long-term prediction of needed financial resources is critical to assure progress towards universal access. [8] While several ‘global price tags’ have been published on the costs to reach the MDGs,[8]–[11] none to date has explicitly examined the resources required to scale up health services to adolescents in low income countries based on bottom-up country by country costing.

The objective of the cost analysis described here, was to estimate the additional resources required to scale up health service interventions with the objective to reduce mortality and morbidity among individuals aged 10 to 19 years in 74 low- and middle- income countries. The results are indicative estimates of the additional investments required to scale up health service delivery above estimated current levels of coverage and quality of care. To our knowledge no study to date has looked at how much countries are currently spending on adolescent. Costs are calculated by country and year, from 2011 to 2015, in order to obtain an aggregated “global price tag” of the incremental financial resources necessary.

## Methods

The following section summarizes the methods used for the cost estimation. For further details on underlying assumptions and measures of the model, see the technical report. [12].

The development of a price tag required the selection of countries and priority interventions, estimation of the population in need of each intervention, and of current and target coverage of interventions, specification of country specific investments associated with the delivery of interventions, and their estimated prices.

### Countries Included in the Costing

Countries included were those defined as having a high disease burden for maternal, child and reproductive health, as included in the “Countdown to 2015 - Maternal, newborn and child Survival” (68 countries) and/or low income countries as defined by the UN Secretary General’s list of least developed countries in 2009 (49 countries), resulting in a total of 74 countries.[13]–[14] (South Sudan is also a high burden country but since data is scarce, South Sudan was not included in the analysis.) These are countries with high rates of HIV prevalence, which in addition to maternal mortality, are key risk factors and outcomes which the selected interventions target. The 74 countries included have a total adolescent population aged 10–19 years, of approximately 954 million in year 2015. The list of countries included and selected country-specific characteristics is provided in Table 1.

**Table 1:** Countries, WHO regions, adolescent population, HIV prevalence and sexual activity.

Country	WHOregion¥	Population of adolescents, 2010 (thousands)	HIV prevalence (%) [18] ^18^		Sexual activity 10–14 years (%) [25] ^25^		Sexual activity 15–19 years (%) [25] ^25^	
			Boys	Girls	Boys	Girls	Boys	Girls
Angola	AFR D	4 523	0,24	0,48	8,94*	17,80*	39,47*	61,95*
Benin	AFR D	2 100	0,12	0,24	13,10	12,30	47,40	53,60
Botswana	AFR E	431	2,49	4,30	14,32	13,77	45,96	54,70
Burkina Faso	AFR D	3 734	0,33	0,41	3,80	7,20	31,80	62,00
Burundi	AFR E	1 958	0,75	1,02	14,32	13,77	45,96	54,70
Cameroon	AFR D	4 520	0,62	1,29	11,10	19,60	50,40	68,20
Central African Republic	AFR E	1 034	0,82	1,07	14,32	13,77	45,96	54,70
Chad	AFR D	2 696	0,35	0,70	10,20	26,30	37,60	74,70
Comoros	AFR D	144	0,14	0,14	8,94*	17,80*	39,47*	61,95*
Congo	AFR E	858	0,60	0,99	26,90	23,50	74,30	80,90
Cote d’Ivoire	AFR E	4 892	0,61	0,82	15,40	19,30	54,30	70,80
Democratic Republic of the Congo	AFR E	16 410	1,32*	2,30*	14,32	13,77	45,96	54,70
Equatorial Guinea	AFR D	158	0,63	1,39	8,94*	19,30*	54,30*	70,80*
Eritrea	AFR E	1 135	0,18	0,18	14,32	13,77	45,96	54,70
Ethiopia	AFR E	20 466	1,32*	2,30*	1,70	15,80	14,10	48,60
Gabon	AFR D	344	0,58	1,11	8,94*	13,77*	45,96*	54,70*
Gambia	AFR D	399	0,25	0,65	8,94*	13,77*	45,96*	54,70*
Ghana	AFR D	5 420	0,25	0,45	4,30	7,80	26,80	41,20
Guinea	AFR D	2 357	0,21	0,35	17,10	21,90	54,40	69,70
Guinea-Bissau	AFR D	365	0,27	0,55	8,94*	17,80*	39,47*	61,95*
Kenya	AFR E	9 213	1,17	1,79	28,80	13,70	61,20	48,10
Lesotho	AFR E	517	2,63	5,03	13,10	6,40	48,90	38,00
Liberia	AFR D	946	0,25	0,34	8,50	17,20	53,60	79,80
Madagascar	AFR D	4 803	0,04	0,04	8,60	15,00	52,70	58,70
Malawi	AFR E	3 888	1,63	2,54	13,70	14,80	47,70	57,10
Mali	AFR D	3 158	0,16	0,223	5,40	24,70	27,40	73,00
Mauritania	AFR D	752	0,13	0,14	8,94*	17,80*	39,47*	61,95*
Mozambique	AFR E	5 394	0,96	2,34	26,40	28,00	64,10	78,70
Niger	AFR D	3 646	0,09	0,15	5,00	29,70	22,70	72,90
Nigeria	AFR D	36 152	0,55	1,01	5,70	15,70	25,60	47,80
Rwanda	AFR E	2 245	0,51	0,61	13,20	3,90	26,30	19,10
Sao Tome and Principe	AFR D	40	0,29*	0,56*	8,94*	17,80*	39,47*	61,95*
Senegal	AFR D	3 073	0,08	0,19	12,40	9,40	37,90	36,90
Sierra Leone	AFR D	1 296	0,16	0,32	11,00	24,60	44,80	66,80
South Africa	AFR E	9 979	1,64	4,22	14,32	13,77	45,96	54,70
Swaziland	AFR E	309	2,32	4,81	4,80	6,90	36,70	46,30
Togo	AFR D	1 552	0,36	0,72	8,94*	17,80*	39,47*	61,95*
Uganda	AFR E	8 334	1,17	1,88	12,20	15,50	49,90	64,20
United Republic of Tanzania	AFR E	10 275	0,91	1,49	9,40	12,40	43,20	62,50
Zambia	AFR E	3 179	1,94	3,10	16,00	13,50	50,60	59,80
Zimbabwe	AFR E	3 291	2,67	3,65	4,50	5,30	26,20	37,00
Bolivia	AMR D	2 213	0,04	0,05	12,50	7,00	60,00	40,10
Brazil	AMR B	33 821	0,12*	0,16*	27,60	10,80	68,20	46,75
Guatemala	AMR D	3 389	0,12	0,09	27,60	10,80	68,20	46,75
Haiti	AMR D	2 298	0,35	0,57	42,70	14,60	76,40	53,40
Mexico	AMR B	20 915	0,04	0,04	27,60	10,80	68,20	46,75
Peru	AMR D	5 822	0,06	0,04	27,60	10,80	68,20	46,75
Afghanistan	EMR D	7 018	0,13*	0,27*	8,94±	13,50±	39,47±	53,40±
Djibouti	EMR D	200	0,50	1,01	8,94±	13,50±	39,47±	53,40±
Egypt	EMR D	16 543	0,01	0,01	8,94±	13,50±	39,47±	53,40±
Iraq	EMR D	7 395	0,13*	0,27*	8,94±	13,50±	39,47±	53,40±
Morocco	EMR D	6 210	0,03	0,03	8,94±	13,50±	39,47±	53,40±
Pakistan	EMR D	40 753	0,02	0,01	8,94±	13,50±	39,47±	53,40±
Somalia	EMR D	2 088	0,11	0,19	8,94±	13,50±	39,47±	53,40±
Sudan	EMR D	9 936	0,13	0,39	8,94±	13,50±	39,47±	53,40±
Yemen	EMR D	6 073	0,13*	0,27*	8,94±	13,50±	39,47±	53,40±
Azerbaijan	EUR B	1 570	0,01	0,07	0,60	0,50	22,10	11,50
Kyrgyzstan	EUR B	1 116	0,02	0,02	8,94±	13,50±	39,47±	53,40±
Tajikistan	EUR B	1 694	0,01	0,01	8,94±	13,50±	39,47±	53,40±
Turkmenistan	EUR B	1 043	0,01*	0,03*	8,94±	13,50±	39,47±	53,40±
Uzbekistan	EUR B	5 972	0,01	0,01	8,94±	13,50±	39,47±	53,40±
Bangladesh	SEAR D	33 976	0,00	0,00	3,15	8,80	19,45	46,70
Democratic People’s Republic of Korea	SEAR D	3 940	0,04*	0,04*	3,15	8,80	19,45	46,70
India	SEAR D	244 515	0,04	0,04	2,30	10,10	11,20	43,00
Indonesia	SEAR B	40 919	0,01	0,01	3,15	8,80	19,45	46,70
Myanmar	SEAR D	8 838	0,12	0,11	3,15	8,80	19,45	46,70
Nepal	SEAR D	6 929	0,06	0,05	4,00	7,50	27,70	50,40
Cambodia	WPR B	3 534	0,12	0,13	0,30	0,90	8,00	19,00
China	WPR B	200 668	0,07*	0,08	1,00	0,97	9,60	14,80
Lao People’s Democratic Republic	WPR B	1 585	0,06	0,06	1,00	0,97	9,60	14,80
Papua New Guinea	WPR B	1 563	0,12	0,17	1,00	0,97	9,60	14,80
Philippines	WPR B	19 870	0,00	0,01	2,40	1,50	17,50	14,90
Solomon Islands	WPR B	121	0,07*	0,08*	1,00	0,97	9,60	14,80
Viet Nam	WPR B	16 815	0,01	0,01	0,30	0,50	3,30	10,50

¥ AFR D (African Region D), AFR E (African Region E), AMR B (Region of the Americas B), AMR D (Region of the Americas D), EMR D (Eastern Mediterranean Region D), EUR B (European Region B), SEAR B (South-East Asia Region B), SEAR D (South-East Asia Region D) & WPR B (Western Pacific Region B).

* Regional average was applied

± Global average of all 74 countries was applied.

### Health Care Interventions Included in the Costing

A standard set of effective priority interventions were chosen based on expert opinion of WHO staff in combination with WHO guidelines on interventions and strategies to address the major causes of burden of disease among adolescents in these countries, with a focus on reproductive health.[15]–[17] Interventions were classified into four packages including a preventive essential package, a preventive expanded package, a curative essential package and a curative expanded package. The essential packages contain those interventions considered to be of potential necessity to a larger number of adolescents than the expanded packages. Identified interventions were assumed to be implemented in all 74 countries with adjustments made for different epidemiological circumstances.

Interventions were assumed to be delivered at three delivery points including hospital care, health facility care and the community (by a health worker or peers). The service delivery distribution of interventions across delivery points was assumed to remain constant during the scale-up period.

Activities carried out in other sectors that may contribute to improved adolescent health are not included in the analysis, such as nutrition interventions, access to education, or structural interventions such as changes in laws and policies.

### Population in Need

The age group of the analysis is adolescents aged 10–19 years (where applicable, further categorized into 10–14 and 15–19 years, and by sex). The population in need was identified per intervention and estimated based on population size (UN Populations Division’s 2008 Revision projections) and incidence or prevalence of a condition.

Data on incidence or prevalence of a condition or risk was based on various data sources.[18]–[25] When country-specific data was available, this was used. For example country-specific HIV prevalence [20] was applied whereas regional incidence data for STIs as per WHO regions [19] was used to determine the population in need for STI management.

The impact of scaling-up interventions on rate of disease occurrence was not accounted for and the model assumes that epidemiological risk will remain constant until 2015. The estimated need for health services is therefore only affected by population growth in the model.

A list of interventions, delivery points and population in need is provided in Table 2.

**Table 2:** Health service interventions to improve adolescent health and their population in need, coverage and points of delivery as estimated in model.

Interventions Ω	Population in need		Current coverage		Delivery points		
	Source of data	Range of estimates used	Source of dataβ	Range of estimates used	Hospital	Primary facility	Community/outreach
**Preventive essential package**							
**Contraceptive services**							
1a.Information	Expert opinion*	19–99%	Expert opinion*	2–30%		90%	10%
1b.Contraceptive counseling & provision	DHS 2000–2010	0–81%	DHS 2000–2010	0–75%		95%	5%
1c.Condom distribution	DHS 2000–2010	0–81%	DHS 2000–2010	0–69%		50%	50%
**Maternity care**							
2a. Basic antenatal care	DHS 2000–2010, Neal et al	3–29% of female adolescents	GHO, 2010	0–98,8%	90%	5%	5%
2b. Care during childbirth	DHS 2000–2010,Neal et al	3–29% of female adolescents	GHO, 2010	98,8%	25%	75%	
2c. Postpartum and postnatal care	DHS 2000–2010,Neal et al	3–29% of female adolescents	GHO, 2010	98,8%		60%	40%
2d. PMTCT	DHS 2000–2010,Neal et al	0–1% of female adolescents	GHO, 2010	98,8%	20%	80%	
**HTC**							
3. HTC	Expert opinion±	0–81%	DHS 2000–2010	0–35%	80%	20%	
**Curative essential package**							
**Management of STIs**							
4a. Syphilis	WHO, 2005	0–5%	DHS 2000–2010	0–81%	80%	20%	
4b. Gonorrhoea	WHO, 2005	0–8%	DHS 2000–2010	0–81%	80%	20%	
4c. Chlamydia	WHO, 2005	1–5%	DHS 2000–2010	0–81%	80%	20%	
4d. PID	Expert opinion*	2–4% of female adolescents	DHS 2000–2010	0–81%	80%	20%	
**Safe abortion services**							
5a.Safe abortion care	Expert opinion¥	1–4% of female adolescents	Expert opinion¥	0–95%	80%	20%	
5b. Post abortion care	Expert opinion¥	1–4% of female adolescents	Expert opinion¥	0–75%	20%	80%	
**Preventive expanded package**							
**HIV harm reduction**							
6a. Needle & syringe exchange	UN Reference Group	0–1%	Mathers et al, 2010	0–81%		30%	70%
6b. OST	Expert opinion∞	0–0,5%	UN Reference Group	0–67%		100%	
**Curative expanded package**							
**Care of injuries due to IPV**							
7. Injury care due to IPV	Termin et al, 2009	0–12% of female adolescents	DHS 2004	0–1%	70%	20%	10%
**HIV care and treatment**							
8a.Care, support & treatment of opportunistic infections	Expert opinion ±	0–3%	UNAIDS	0–58%			
8a. ART provision	Expert opinion ±	0–1%	UNAIDS Global Epidemics Report	17–83%	85%	15%	

* Jane Ferguson, Chandra Mouli-V, Department of Maternal, Newborn, Child and Adolescent Health, WHO.

± Jesus Maria Garcia Calleja, Technical Officer & Rachel Baggaley Medical Officer, HIV Department, WHO.

¥ Elisabeth Åhman, formerly WHO staff.

∞ Annette Verster, Technical Officer, HIV Department, WHO.

β DHS (Demographic Health Survey), GHO (Global Health Observatory) & UNAIDS (the Joint United Nations Programme on HIV/AIDS).

Ω PMTCT (Preventing Mother-To-Child Transmission (of HIV), PID (Pelvic Inflammatory Disease), HTC (HIV testing and counseling, OST (Opioid Substitution Therapy), IPV (Intimate Partner Violence) & ART (Antiretroviral Therapy).

### Estimating Current Coverage Levels

Coverage was defined as the proportion of adolescents receiving the interventions from those in need. The target for scale-up was set at 95% - as per other ‘universal coverage’ targets that have been costed, [26] - except for opioid substitution therapy, which was set at 50% in accordance with WHO guidelines. [27] Country specific current coverage levels were estimated using a variety of data sources.[25], [28]–[29] For most interventions, this entailed using data from Demographic and Health Survey (DHS), most recent year from 2000 or later. For countries for which there was no coverage data available, regional averages were applied (population weighted when possible). For interventions for which no DHS or other data was available, experts within WHO and UNAIDS were consulted.

The incremental scale-up of coverage required was estimated using the target of universal access (95%) minus the current coverage levels per country and intervention. We also ran the models with 0% starting coverage so as to obtain an estimate for resources required to scale-up from 0 to 95%. Subtracting the difference allowed us to estimate what resources may currently be spent on adolescent health.

### Programme Activities for Adolescent-friendly Health Services

Programme activity costs are expenses incurred at the levels of facility, district or national level, [30] and here refer to improving the management and quality of care provided to adolescents. Programme activities were identified based on the implementation of an approach which seeks to advance the adoption of characteristics related to the dimensions of WHO quality of care.[31]–[33] To be considered adolescent-friendly, services should be *equitable, accessible, acceptable, appropriate* and *effective*. Based on these five broad dimensions of quality, 20 characteristics were used to identify and define programme activities required to improve service delivery (table 3). Programme activities were classified into programme management, training, supervision, information, education and communication (IEC), infrastructure and equipment.

**Table 3:** Characteristics of Adolescent Friendly Health Services.

Nr.	Characteristic
**Equitable**	
**1**	Policies and procedures are in place that do not restrict the provision of health services on any terms
**2**	Health care providers treat all adolescent clients with equal care and respect, regardless of status
**3**	Support staff treats all adolescent clients with equal care and respect, regardless of status.
**Accessible**	
**4**	Policies and procedures are in place that ensure that health services are either free or affordable to adolescents.
**5**	The point of health service delivery has convenient hours of operation.
**6**	Adolescents are well-informed about the range of available reproductive health services and how to obtain them
**7**	Community members understand the benefits that adolescents will gain by obtaining the health services they need, and support their provision.
**8**	HF Some health services and health-related commodities are provided to adolescents in the community by selected community members, outreach workers and adolescents themselves.
**Acceptable**	
**9**	Policies and procedures are in place that guarantee client confidentiality.
**10**	The point of health service delivery ensures privacy.
**11**	Health-care providers are non-judgmental, considerate, and easy to relate to.
**12**	The point of health service delivery ensures consultations occur in a short waiting time, with or without an appointment, and (where necessary) swift referral.
**13**	The point of health service delivery has an appealing and clean environment.
**14**	The point of health service delivery provides information and education through a variety of channels.
**15**	Adolescents are actively involved in designing, assessing and providing health services.
**Appropriate**	
**16**	The required package of health care is provided to fulfill the needs of all adolescents either at the point of health service delivery or through referral linkages.
**Effective**	
**17**	Health-care providers have the required competencies to work with adolescents and to provide them with the required health services.
**18**	Health-care providers use evidence-based protocols and guidelines to provide health services.
**19**	Health-care providers are able to dedicate sufficient time to work effectively with their adolescent clients
**20**	The point of health service delivery has the required equipment, supplies, and basic services necessary to deliver the required health services.

Source: From reference [32] WHO (2009) Quality assessment guidebook – a guide to assessing health services for adolescents.

### Costing the Scale-up

Costs are classified as *intervention costs* or *programme activity costs* (Table 4). Intervention costs refer to direct costs of delivering services to the patient and include drugs, laboratory tests, medical supplies and service delivery costs such as consultation time based on guidelines and recommendation of health care delivery.[18], [20], [34]–[39].

**Table 4:** Activities and components of intervention- and programme activity costs of adolescent friendly health services.

Cost category	Components/Activities	Costs included
**Intervention costs**		
Commodities	Drugs, vaccines, laboratory tests, medical supplies	Drugs, vaccines, laboratory tests, medical supplies
Service delivery costs	Costs for health care visits including salaries of health workers and locally procured goods, such as overhead costs for electricity, water and buildings	Costs for health care visits including salaries of health workers and locally procured goods, such as overhead costs for electricity, water and buildings
**Programme activity costs**		
Programme management	General programme coordination at national- and district level, assessment and revision of existing policy and the development, production and distribution of national standards for AFHS	Per diems, travels, support services
Training	Training of professional health workers at facility- and hospital level, and for community workers providing outreach services. Adaptation of pre-service curriculum	Per diems, travels, room rental, refreshments, training material
Supervision	Supervision at district- and facility level and of community health workers	Per diems, travels
Information, communication & education	Development of IEC materials (posters, radio and billboards at national- and district level) & sensitization sessions at facility level	Billboards, radio adverts, posters, leaflets, beverages, room rental
Infrastructure & Equipment	Upgrade of infrastructure and equipment to standards at facility level	Screen walls, secure cabinets, paint & decoration supplies

For programme costs, the quantities of investments were determined based on the WHO Quality of Care model and discussions with the WHO staff. Details of programme activities as well as underlying assumptions of interventions are provided in the technical report. [12].

Three different kinds of models were used. To estimate costs for the majority of the interventions, a new costing model was created containing assumptions for all interventions on coverage rates, population in need, and ingredients of health care. For maternity care, an existing model was used with adaptation to adolescent population data and updated prices. [40] Note that 6 of the 74 countries were not included in this model and therefore costs for maternity care have not been included for the following countries: Botswana, Democratic People’s Republic of Korea, Sao Tome and Principe, Solomon Islands, Turkmenistan, and Uzbekistan. For programme costs, a new costing model was created containing assumptions for components of programme activities. Somalia was excluded from the programme activity costing due to lack of country-specific data.

The models use a standardized ingredients approach (cost = quantity x price) to generate country specific cost estimates. [41] Costs are estimated for each country as the number of cases multiplied by the cost per case. The number of cases was based on epidemiological and demographic data and coverage rates. Cost per case is based on intervention component quantities times the unit price of components, using country-specific prices when available. The scale-up trajectory to 2015 is assumed to be linear for all countries.

Prices (in US dollars) of programme activity inputs such as per diem rates, travel costs, room rentals, and mass media distribution costs were taken from the WHO-CHOICE database. [42] Prices of drugs, laboratory tests and medical supplies were based on median prices reported by Management Sciences for Health’s International drug price guide. [43] Costs for service delivery (inpatient days, outpatient visits) were based on unit costs derived from WHO-CHOICE database. [42].

Costs are presented in US dollars (2008), and estimated by cost category, country and year.

## Results

The cost estimates indicate the additional financial resources needed to scale up health service interventions to adolescents towards universal coverage levels. The 5-year total cost of scale up is estimated at US$ 15.41 billion, of which US$ 1.86 billion in 2011, increasing to US$ 4.31 billion in 2015. This corresponds to approximately US$ 1.02 per adolescent in 2011, increasing to $4.70 in 2015.

**Figure 1:**
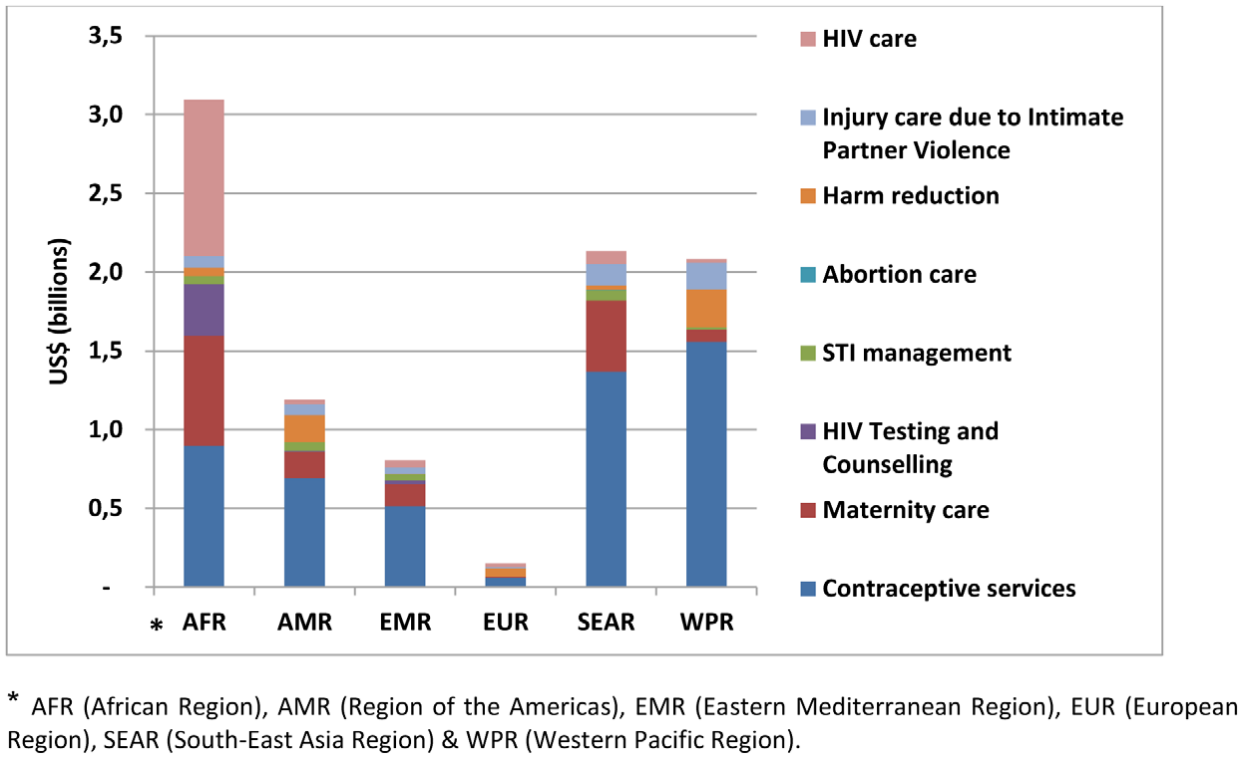
Estimated incremental cost of scaling-up health services to adolescents per intervention and region (total for 74 countries, billion US$ 2008).

Figure 1 shows the estimated incremental cost per intervention and region. Contraceptive services account for the largest investment in all regions, except for the African region where costs for HIV care are slightly larger.

**Figure 2:**
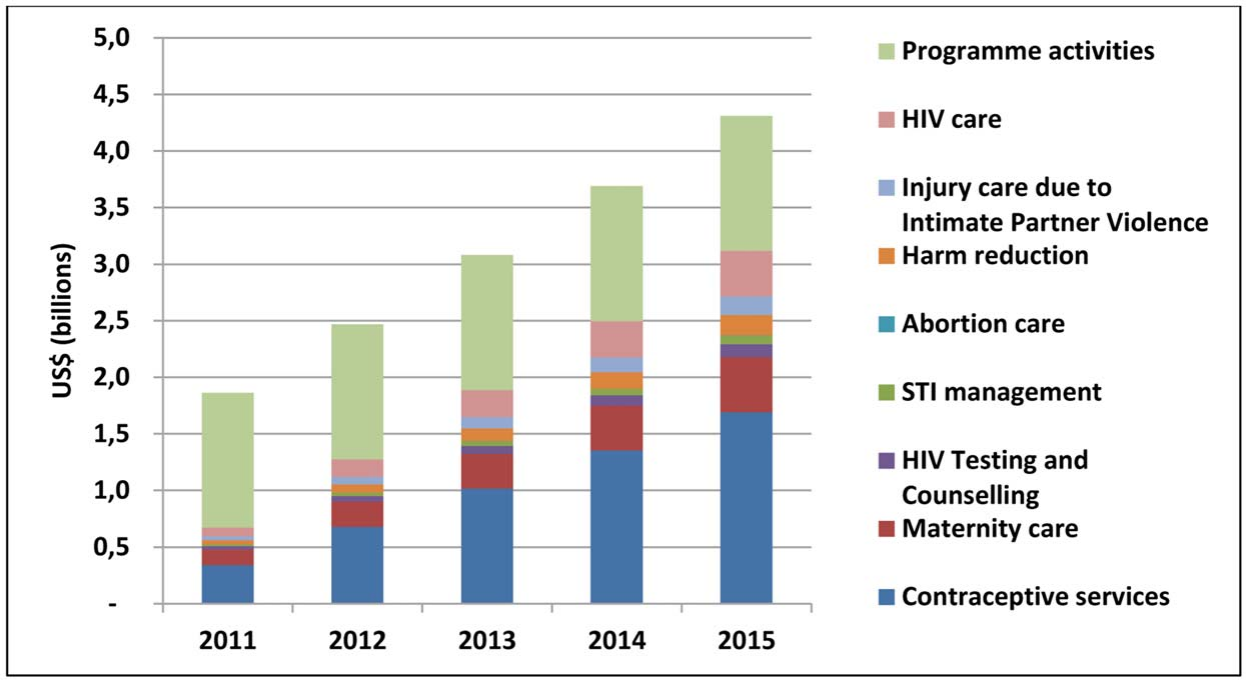
Estimated incremental cost of scaling-up health service delivery to adolescents per intervention set and year (total for 74 countries, billion US$ 2008).

Figure 2 shows the breakdown of costs for each set of interventions per year. The greatest share of the cost is generated by scaling-up programme activities (38.69%), contraceptive services (33.01%), maternity care (10.04%) and HIV care and treatment (7.74%). The cost for STI management, HIV testing and counseling (HTC), abortion care, harm reduction and injury care is smaller, mainly due to regional differences in disease epidemiology (HTC) and legality (abortion and harm reduction).

Initially, the majority of additional investments would be for implementing programme activities: US$ 1.32 billion out of the 1.72 billion in year 2011. By 2015, the cost of direct service delivery and commodities constitute the main cost, US$ 2.64 billion of the US$ 3.83. During the 5 years, 23.12% of all additional costs are accounted for by medicines and commodities. Service delivery costs and commodity costs increase more over time than programme costs due to increased coverage and populations while programme activity costs are modeled to successively increase over time i.e. similar numbers of health workers are projected to be trained by year, and only the IEC component of programme activity costs is dependent on population size.

Costs associated with the programme activities are important expenditures to improve quality [44] that is necessary to address barriers adolescents face in accessing health services. Costs were estimated at US$ 5.96 billion, remaining relatively constant at 1.19 billion per year. This corresponds to US$ 1.26 per adolescent and year.

**Figure 3:**
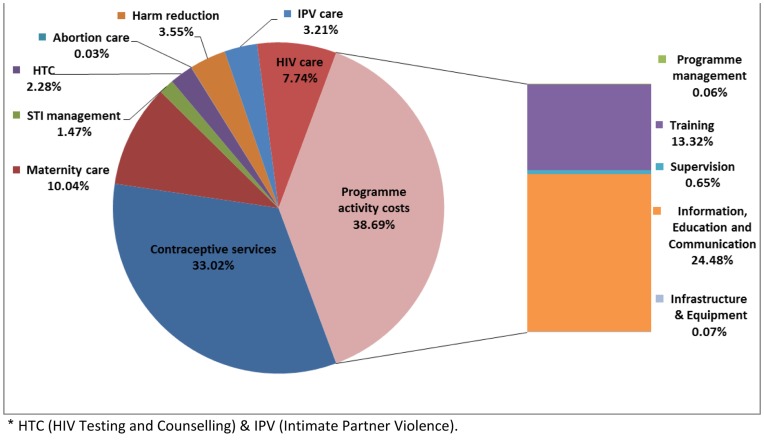
Estimated incremental costs per intervention as percentage of total costs.

Figure 3 shows the breakdown of costs by intervention. The largest cost category is programme activities, of which IEC is the largest cost, accounting for 24.48%. Contraceptive services and maternity care are the most costly interventions, accounting for 33.02% and 10.04% of total costs, respectively, followed by HIV care and treatment (7.74%). The population in need for contraceptive services and maternity care is proportionately larger than for the other interventions while HIV care and treatment generates high commodity costs. Other interventions represent 3.55% or less of the total estimated cost. In the 35 low income countries with 19% of the population in need and currently spending on average (population weighted) 23.80 per capita [45] on total health expenditure, 2.94 billion would be needed. In the 39 middle income countries with 81% of the population and with current average total health spending of 136.76 per capita, [45] 12.47 billion would be needed until 2015.

## Discussion

This study provides, for the first time, the estimated resource requirements of scaling-up the coverage and quality of health service delivery to adolescents in high burden countries. Results indicate a global additional cost of approximately US$ 15.41 billion or 3.08 billion per year on average. Running the model from 0% coverage indicates that current spending on the key set of adolescent services approximates US$ 1.49 billion per year on average, which means the additional investment would require a three-fold increase. In comparison with total health expenditure (THE) per capita, the additional need is less substantial – the estimated AFHS-related costs in 2015 would be equivalent to 0.72% increase above current (2009) THE. [45] The greatest investment need is in the African region, but also in the Western Pacific and South East Asian regions, and concern contraceptive services, maternity care and HIV treatment and care. These estimates are indicative, as context-specific factors would influence the expansion of services at country level.

This paper does not consider financing scenarios. However, it should be noted that in the short run the public sector may not be able to bear the full costs for expanding coverage and there may be need for investment from private sector actors as well as investments by external donors where appropriate. Two important points can be made however. First, in view of recent findings that Official Development Assistance for Family Planning is decreasing [46] our finding that contraceptive services represent 33% of the additional costs underlines the need to raise new resources to support this important area. This was further emphasised at the recent Family Planning Summit, where pledges of $4.6 billion were made for improving delivery of family planning services from 2013–2020. Our estimate is higher ($5.1 billion 2011–2015), but includes a more extensive definition of contraceptive services, scaled up to achieve universal coverage. Second, the fact that AFHS programme activities constitute 39% of costs highlights the need for countries to invest in quality improvement for health services for adolescents, an area which few countries are investing in. Recent efforts by countries [47] to explicitly include adolescent health in their national strategic health plans demonstrate important progress however. [48].

### Limitations

Cost estimates aim at reflecting WHO’s recommended clinical guidelines, protocols and quality framework to the largest extent possible. However, assumptions have been applied to enable estimates of the nature and extent of health conditions and current coverage.

The impact on disease epidemiology of scaling-up was not accounted for in the estimation of costs. Ideally, improved health services would influence health care need and the changing need over time would be included. However, considering the five year time horizon, it is acceptable to assume limited effect on population epidemiology.

The feasibility of scale-up largely depends on the country specific health care context. The different health sector characteristics and capacities of specific countries were not considered in this exercise. Consequently, programme activities and interventions were assumed to be implemented in all countries, but with consideration of legal restrictions.

The analysis took a provider perspective and does not account for opportunity or time costs for patients, or other implications related to the demand side of a health care analysis.

Estimated costs are dependent on the size of the population in need, current coverage levels and unit prices of service delivery, all of which are based on data from the United Nations Population Division, WHO databases, DHS and WHO expert opinion, and are further described in the technical report. [12] The estimations are largely based on country-specific data but are complemented with regional as well as global estimates when needed.

All three models used the same bottom-up and ingredients-based approaches and hence, coherence of the models was determined to be acceptable.

In summary, scaling up quality health service delivery to adolescents would require less than 1% increase in average total health expenditure but could have enormous benefits to address the 5.6 million DALYs among males and females adolescents in Africa. [3] The emphasis needs to be on quality of care (technical and user friendliness) for the provision of effective interventions.

An important step at country level will be national strategic plans that take into account adolescent health needs and appropriate service delivery. Strategic planning tools such as the OneHealth tool, recently developed by the UN inter-agency working group on costing can help in development of such costed plans. [49] Efforts should be made to improve our estimates of programme activity costs for adolescent friendly health services and ensure that available health sector planning tools include activities to improve adolescent health.
